# Trade routes and plague transmission in pre-industrial Europe

**DOI:** 10.1038/s41598-017-13481-2

**Published:** 2017-10-11

**Authors:** Ricci P. H. Yue, Harry F. Lee, Connor Y. H. Wu

**Affiliations:** 1 Department of Geography, The University of Hong Kong, Hong Kong, China; 2 International Center for China Development Studies, The University of Hong Kong, Hong Kong, China; 3 Department of Population Health Sciences, Virginia-Maryland College of Veterinary Medicine, Virginia Tech, Virginia, USA

## Abstract

Numerous historical works have mentioned that trade routes were to blame for the spread of plague in European history, yet this relationship has never been tested by quantitative evidence. Here, we resolve the hypothetical role of trade routes through statistical analysis on the geo-referenced major trade routes in the early modern period and the 6,656 geo-referenced plague outbreak records in AD1347–1760. Ordinary Least Square (OLS) estimation results show that major trade routes played a dominant role in spreading plague in pre-industrial Europe. Furthermore, the negative correlation between plague outbreaks and their distance from major trade ports indicates the absence of a permanent plague focus in the inland areas of Europe. Major trade routes decided the major plague outbreak hotspots, while navigable rivers determined the geographic pattern of sporadic plague cases. A case study in Germany indicates that plague penetrated further into Europe through the local trade route network. Based on our findings, we propose the mechanism of plague transmission in historical Europe, which is imperative in demonstrating how pandemics were spread in recent human history.

## Introduction

Plague is initiated by the flea-borne bacterium *Yersinia pestis*, which circulates mainly on rodents and other mammal hosts through the rodent’s associated fleas^1,2^. Normally, the bloodsucking fleas acquire *Yersinia pestis* from an infected rodent. The bacterium will quickly multiply and cluster, leading to the blockage of the alimentary canal in the fleas’ guts^3^. When the infected flea jumps onto another mammal, preferably rodents, it will transmit the bacteria to the new host by regurgitating the clotted blood from the blockage of the alimentary canal^4^. If an infected flea attempts to feed on a human, it will transmit *Yersinia pestis* to the human and lead to human plague in the form of bubonic plague or pulmonary plague. Traditional thought suggested that the clustering of *Yersinia pestis* rarely happened on human fleas^5^. Yet, recent evidence revealed that not only rodent fleas (*Xenopsylla cheopis* as a classic example) are to blame for plague transmission, human fleas (*Pulex irritans*) and cat fleas (*Ctenocephalides felis*) are also likely to play a role in disseminating *Yersinia pestis* ^6,7^. Furthermore, laboratory results illustrated the experimental possibility of oral route transmission of plague^8^ and epidemiological records suggested plague infection through consumption of contaminated meat^9,10^.

Despite improvements in sanitation and medical advancements in the course of human history, plague remains a major threat to human beings^11,12^. Over the past few decades, thousands of cases of human plague have occurred around the world, particularly in Africa^13^. Some researchers suggested that plague may reign over our planet again when global climate change makes some places on earth become wetter and hotter^14–16^. Considering the widespread wildlife reservoirs of plague foci, together with the quick spread, rapid clinical course, inherent communicability, and high mortality rate of plague, the risk of plague outbreak should never be underestimated, although the number of human plague cases is relatively low compared to other infectious diseases at present^17^. Flashing back in history, plague caused three great pandemics, in which 200 million people perished^18^. Given the nature of plague and its notorious history, the international community should be more prepared for the re-emergence of plague. Ironically, remarkably little research has been done to elucidate how plague spread through metastatic spatial domains and the mechanism behind its distribution.

The idea that infectious diseases are spread by transportation routes has been supported by proof from various studies on infectious diseases^19–22^. In an earlier study, the authors also hypothesized the possible role of trade routes in plague distribution through examining the linkage between navigable rivers and plague outbreaks^23^. Using the geo-referenced Old World trade route database prepared by Ciolek^24^ and the historical human plague outbreak distribution in Europe by Büntgen *et al*.^25^, this study examined the extent to which major trade routes shaped the dispersal of plague in Europe between AD1347 and 1760. The study timespan was determined by the availability of data before the Industrial Revolution in Europe (see SI Text for details). During the late medieval period in Europe, long-distance maritime trade routes were developed as a result of the commercial revival of Italy^26^. Combined with the already active overland trade routes in Western Europe^27^, they formed a comprehensive network that linked European cities together. Commodity goods, agricultural products, and luxury items were circulated amongst major port cities and the continental hinterland beyond until the Industrial Revolution introduced a new era of trading patterns. It was also a period when the agricultural sectors of Europe had a surplus of labor, and peasants were pushed to the city, generating rapid urban growth^28^. Human movement became more frequent than in previous eras. Under the influence of these conditions, infectious disease gained access and penetrated every corner of Europe. Would it be possible that trade routes was one of the determining factor in shaping the pattern of plague outbreak in historical Europe? We sought to answer this question in the present study.

Other scholars have long identified the influence of trade routes on plague transmission in historical Europe^20,23,29,30^. Yet, no scientific consensus could be reached concerning the coherence of plague outbreaks and major trade route patterns. Here, we based on our statistical results to prove that there would be more plague outbreaks when a city is closer to the major trade routes. Moreover, the evidence found in our investigation of the plague/trade-port relationship did not indicate any sign of permanent plague reservoirs in the inland areas of Europe in history. Plague was imported from trade ports or it originated from somewhere linked to the maritime trading system. Sporadic plague outbreak was used as an indicator to show the different roles of trade routes and navigable rivers in plague transmission. A specific case study of Germany indicated that a local trade route was more important in distributing plague outbreak. Altogether, the results allowed us to propose a potential plague transmission mechanism.

## Results

**Figure 1:**
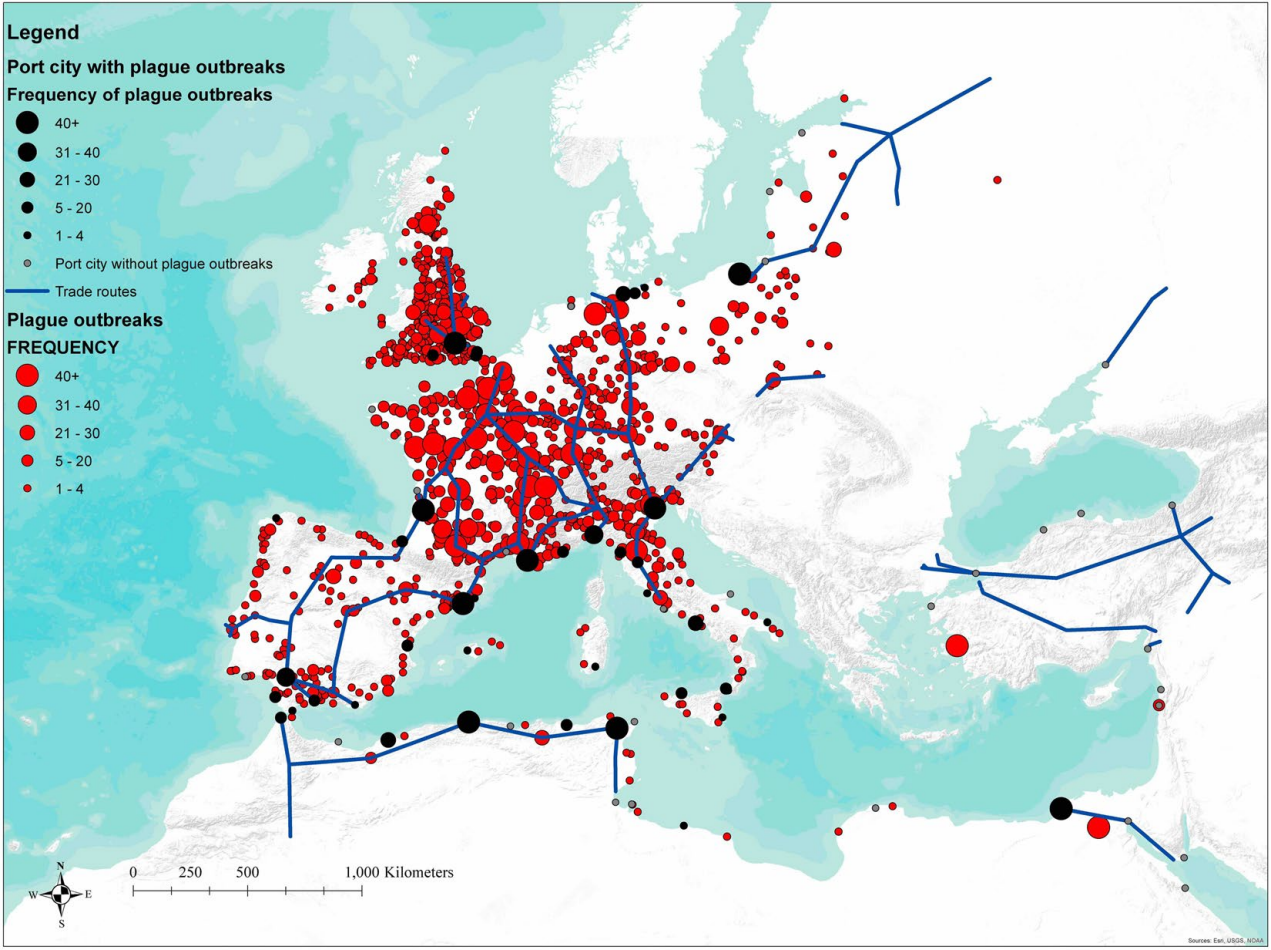
Spatial distribution of plague outbreak in Europe, and Northern Africa, AD1347–1760. Plague outbreaks are related to the patterns of trade routes, both overland and maritime, and also major trade ports in pre-industrial Europe. Cities with recorded plague outbreaks are marked with red dots, with the size of dots referring to the number of plague outbreak during the study period (See legends). The blue lines indicate the major trade route in early modern Europe. The black dots identify the locations of major trade ports with plague outbreak over the study period. Major trade ports without plague outbreak over the study period are labeled in grey dots. Trade routes and trade ports at countries with no plague record are omitted. From our results, more plague outbreaks happened in the periphery of trade routes and trade ports. The map is generated in ArcGIS version 10.1 (www.esri.com/software/arcgis).

We combined records of 6,656 plague outbreak cases in historical Europe and North Africa and the trade route database that geo-referenced the major overland and maritime trade routes during the early modern period (Fig. 1). To examine whether trade routes were related to the plague outbreak patterns during our study period, we started by checking whether plague hotspots were also key trade nodes. According to a recent study by Schmid *et al*.^30^, there was never any permanent plague reservoir in pre-industrial Europe. This implied that a plague outbreak at any given place in our study area was transmitted from a nearby outbreak. If human movements and the circulation of goods provided an ideal channel for the spread of plague, a city’s proximity to key trade nodes would determine its likelihood to become a plague hotspot.

The top 20 cities with the highest year count of plague outbreaks between AD1347 and 1760 are listed in Table 1. Thirteen of these cities were key trade nodes that linked trade routes together. These cities were geographically dispersed and spread in seven countries. As stated by Vogler *et al*.^31^, maritime ports were common plague outbreak centers when “plague ships” introduced infected rodent hosts and flea vectors to the cities. In addition, even though half of the trade nodes as documented in our dataset are port cities, only six port cities (in six countries) are listed in Table 1. The same result was also obtained with a sensitivity test (Table S2). Briefly, plague hotspots were mostly trade nodes. Yet, there seemed to be no evidence to support that those hotspots were necessarily port cities. We dissimilated this pattern by searching for the relationship between plague outbreak and its distance to trade route and trade port.

**Table 1:** List of top 20 plague hotspot cities and their role in old world trade route in Europe, AD1347–1760. Key trade node refers to the major transportation node connecting medieval Europe as indicated by Evans and Brooke^41^ and Spufford^42^. Major port city indicates whether it also functioned in maritime trade route.

Name of City	Count of Year with Plague Outbreak	Key Trade Node	Major Port City
London	132	Yes	Yes
Alger	90	Yes	Yes
Paris	89	Yes	No
Toulouse	88	Yes	No
Bourg-en-Bresse	77	No	No
Amiens	75	No	No
Nantes	73	No	No
Bordeaux	68	Yes	Yes
Venezia	63	Yes	Yes
Strasbourg	59	Yes	No
Rouen	57	No	No
Limoges	57	Yes	No
Basel	56	Yes	No
Angers	55	No	No
Dijon	53	Yes	No
Troyes	52	No	No
Bremen	46	No	No
Barcelona	46	Yes	Yes
Gdansk	44	Yes	Yes
Milan	44	Yes	No

If plague was spread along trade routes, the closer one got to a trade route, the easier it would be to become infected. Therefore, we calculated the distance between each plague outbreak and its closest trade route and estimated its relationship with the accumulated count of each plague outbreak point. Several control variables were included in our Ordinary Least Square (OLS) regressions to investigate the robustness of the relationship. Our results are summarized in Table 2. Model 1 was the base model for estimating the relationship between logged distance to trade route and plague count. The association was negative, which supported our hypothesis. In Model 2, the regression was run with both the time fixed-effects and the region fixed-effects to control the influence of any observable or unobservable predictors over the dependent variable. Hence, bias on omitted variables was greatly reduced. The relationship between logged distance to trade route and plague count remained negatively significant. In Model 3, the regression was run with additional geographical controls. As climate is one of the most dominant factors in determining the prevalence of plague^13,15,16,32,33^, we controlled the effect of temperature on plague reoccurrence by incorporating the elevation and the latitude of plague location into the regression. In addition, the longitude variable also helped to capture the difference in plague distribution between the eastern part and the western part of Europe^34^. The association continued to remain negatively significant. Further control on climatic zone did not affect the negative correlation between plague count and its distance from trade route (Table S1). As there might be difference between North Africa and Europe, as well as between coastal city and inland city, in terms of their plague recurrence, we included a North Africa dummy and a coastal city dummy in Model 4. Our results showed that no significant difference is seen between North Africa and Europe. In parallel, although coastal cities were more prone to plague outbreak, it did not distort the negative correlation between plague count and its distance to trade route. In Model 5, we included vegetation cover and normalized population density, which symbolizes the degree of urban development over different plague outbreak points, as control variables in the regression. In Model 6, we included per capita Gross Domestic Product (GDP), Consumer Price Index (CPI), and normal laborer wages as control variables in the regression. The specifications in Models 5 and 6 were set according to the practices of traditional public health studies. The rapid and unplanned urbanization process would promote the spread of epidemics^35^, while landscape contexts were associated with plague occurrence^36^. On the other hand, accumulated wealth and improvements in the living standard in early modern Europe might dampen plague reoccurrence. Nevertheless, the inclusion of these control variables did not alter the significance and robustness of our estimated relationship.

**Table 2:** OLS estimates of relationship between plague outbreak and trade route in Europe, AD1347–1760.

	Model 1	Model 2	Model 3	Model 4	Model 5	Model 6
Log(Distance from city centre)	−6.086034*** (*β* = −0.4665029)	−6.181281*** (*β* = −0.4738037)	−6.175487*** (*β* = −0.4733596)	−6.289981*** (*β* = −0.4821357)	−6.175596*** (*β* = −0.473368)	−6.174889*** (*β* = −0.4733137)
Latitude			−0.1978809 (*β* = −0.0892771)	−0.0283914 (*β* = −0.070624)	−0.2012694 (*β* = −0.0500661)	−0.203408 (*β* = 0.0505981)
Longitude			1.349016*** ($\beta$ = 0.2379364)	1.212566*** (*β* = 0.2138698)	1.349467*** (*β* = 0.2380161)	1.368032*** (*β* = 0.2412906)
Elevation			−0.0175064*** (*β* = −0.1049527)	−0.023406*** (*β* = −0.1403218)	−0.0174867*** (*β* = −0.1048349)	−0.0174736*** (*β* = −0.104756)
Coast indicator				−9.778023*** (*β* = −0.1281232)		
North Africa Indicator				6.565075 (*β* = 0.0484845)		
Vegetation Cover					0.2457425 (*β* = 0.0913583)	0.3816018 (*β* = 0.1418658)
Normalized Population Density					0023217 (*β* = 0.0699493)	0.0025352 (*β* = 0.0763817)
Per Capita GDP						−0.0101291 (*β* = −0.0857906)
CPI						2.320846 (*β* = 0.0386653)
Normal Wage						0.7168337 (*β* = −0.346733)
Time Fixed effect	No	Yes	Yes	Yes	Yes	Yes
Regional Fixed effect	No	Yes	Yes	Yes	Yes	Yes
Number obs.	6656	6656	6656	6656	6656	6656
R^2^	0.2176	0.4144	0.4339	0.4434	0.4340	0.4345

Notes. The dependent variable of checks is the total number of plague reoccurrence.

***p < 0.005; **p < 0.01; *p < 0.05.

The OLS estimates revealed that distance to trade route has a high explanatory power to the distribution of plague outbreak. Despite its large sample size (n = 6,656), the R^2^ ranged from 0.41 to 0.44 in Models 2 to 6. The negativity of the association implies that cities closer to trade routes were more vulnerable to plague reoccurrence. On the other hand, being further away from trade routes was a good way to escape from plague.

We performed several sensitivity tests to further check the robustness of our OLS regression results (Table S3). It was shown that the relationship between plague outbreak and trade route was highly significant and remained negative in different temporal domains of our study period (AD 1347–1449, AD1450–1549, AD1550–1649, and AD1650–1760). In addition, the relationship was robust in our different specification of spatial domains, in which the cases in Russia and Africa were excluded, or only the cases in continental Europe and the six major plague outbreak countries were included (see SI for more details). The above results implied that the pattern of plague outbreak was determined by the trade route patterns for the entire study area, which was consistent over the study period. Hence, the relationship should be independent of cultural, demographic, economic factors or the possible spatial bias of plague database as suggested by Alfani^37^. Otherwise, the relationship over different regions and time periods should differ.

The pattern of plague outbreak in historical Europe was related to the distribution of major trade routes at that time. However, was plague circulating within villages and cities and being retained as a reservoir, as suggested by Ell^38^? Or was plague repeatedly introduced from major trade ports to inland areas, as suggested by Schmid *et al*.^30^? Here we used the distance to major trade ports as an indicator to determine whether there was a permanent plague focus in historical Europe (Fig. 1). A few plausible scenarios should be considered. If there was a permanent plague focus in historical Europe, plague would circulate between cities and villages, and not necessarily be transmitted through trade ports. Therefore, we should not detect any significant relationship between plague outbreak and distance to trade port. However, there might also be a scenario in which the permanent plague focus was so strong that it kept on exporting plague from inland to other parts of Europe. In this case, the relationship being examined in this section might become positively significant. At the same time, it might be possible that trade ports were always the entrance point or starting point for plague transmission, which moved inland until that wave of plague outbreak was no longer able to sustain itself and faded. In such a case, we should detect a negatively significant correlation between distance to trade port and plague outbreak. The OLS estimation results for the above scenarios were reported in Table S4. It was clear that distance to major trade ports was negatively correlated to plague outbreak (p = 0.000, F = 114.38). The negative relationship highlights that more plague outbreaks were recorded when distance to trade port decreased, suggesting that there should not be any permanent plague reservoir in the inland part of Europe. We also conducted a detailed robustness check on how the pattern of plague outbreak was controlled by the distance to trade ports over different specifications in spatial and temporal domains (Table S5). Overall, the results showed a constantly highly significant and negative relationship between distance to trade port and plague. The relationship was robust and did not vary in any timespan or region that we singled out from the database. This further confirmed the estimation that plague was repeatedly introduced to the inland of Europe through maritime trade ports in our study period. Plague was introduced to Europe mainly by maritime trade routes and entered overland trade routes until that wave of plague outbreak faded away or strengthened again by a new introduction of plague supply. Or it was possible that any of the major ports could be a strong permanent plague reservoir. As Davis^39^ has suggested, in the “upper half” of Europe, the climate was a disadvantage to black rat reproduction. The population of black and brown rats could never be sustained as an epidemic focus without a continuous supply of new rats by ships^40^.

**Figure 2:**
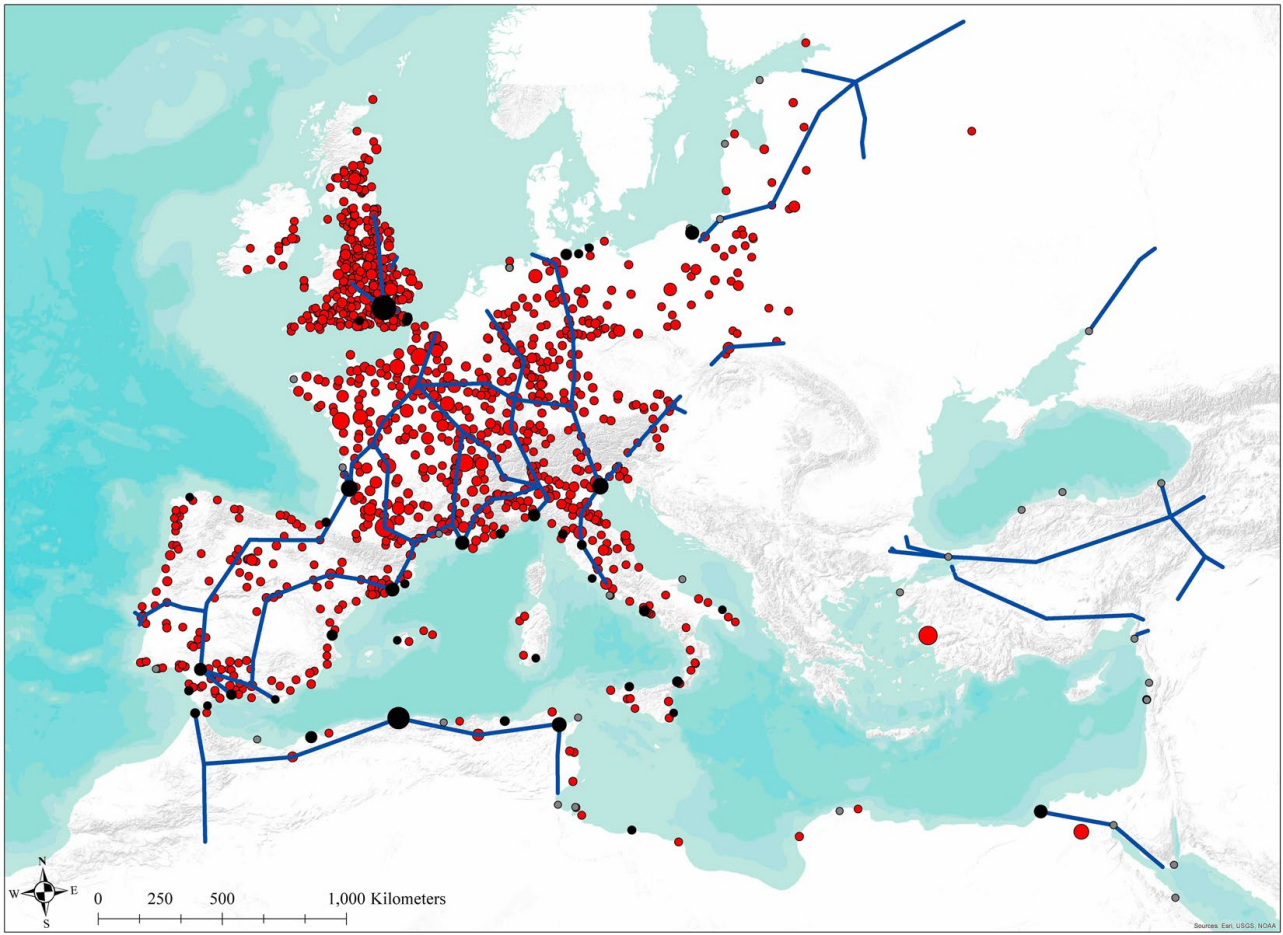
Sporadic plague outbreak (N < 5) did not follow the pattern of major trade routes. The red spots represent the locations of sporadic outbreak of plague (856 locations). The blue lines indicate the major trade route within our study period. The black dots identify the major trade ports with plague outbreak. The trade ports with no reported plague outbreak within our study period are labeled in grey dots. The map is generated in ArcGIS version 10.1 (www.esri.com/software/arcgis).

In the previous analysis, we verified that plagues were repeatedly introduced to Europe through major trade ports, following the trade route to various trade nodes as well as to the cities around the trade routes. This, however, gave rise to another question regarding the extent of the impact of trade route to plague outbreak. Is trade route responsible for shaping all the plague outbreak patterns in historical Europe? To answer this question, we narrowed down our investigation to the sporadic plague outbreak points that had four or fewer outbreaks during our study period (Fig. 2). As shown in Fig. 2, sporadic plague outbreaks (frequency ≤ 4) are scattered around on the map. These sporadic cases did not follow the pattern as described in our previous analysis. The associated statistical result also indicated the relationship between trade routes and plague outbreak to be insignificant (Table S6).

However, there must be certain ways for animal hosts to cause these sporadic outbreaks scattered around the European continent. It might be attributable to some less active transportation routes (e.g., navigable rivers) that connected these sporadic cases with the plague hotspots. We calculated the correlation between distance to the closest navigable river and plague outbreak for these sporadic cases (Table S7). We found that although navigable rivers were not as capable as major trade routes in influencing the total plague transmission (Table S8), they did account for the pattern of sporadic plague distribution in historical Europe (p < 0.005; F = 6.13). There were more outbreaks of plague in those cities located closer to the navigable rivers. The result was also verified by robustness checks in various geographical specifications. It confirmed that local river channels, instead of major trade routes, were more significant in determining the distribution of sporadic plague outbreak cases in early modern Europe.

**Figure 3:**
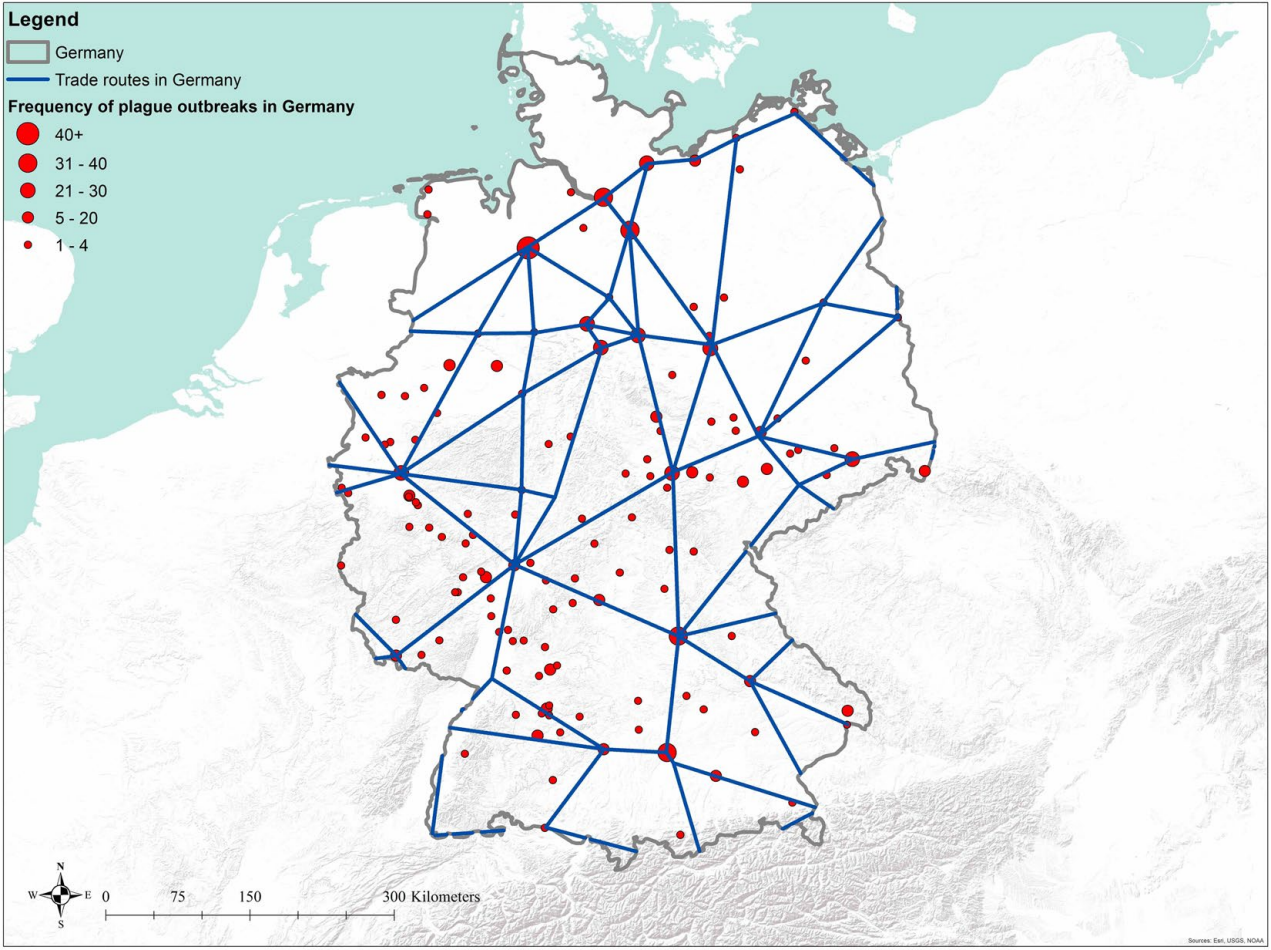
Distribution and frequency of plague outbreak in relation to the local Holy Roman Empire trade route in Germany, AD1347–1760. It can be seen that locations with more plague recurrence (as referred by the size of red dots) are closer to the local Holy Roman Empire trade route (blue lines). The strength of recurrence fades in according to the distance away from these trade routes as suggested by the statistical analysis (Table 3). The map is generated in ArcGIS version 10.1 (www.esri.com/software/arcgis).

The major trade route database by Evans and Brooke^41^ and Spufford^42^ might have neglected the trade route-plague relationship at the local level, as they primarily address the major trade routes and trade ports at the continental scale. Here, we employed a very fine-gained trade route database in Germany, which consists of both major and local trade routes (Fig. 3), to see whether our findings about the trade route-plague relationship could be supported. Our results showed that the parts of Germany that were closer to trade routes, measured by logged distance, tended to be the areas suffering repeated plague occurrence (Model 2 in Table 3). Also, we repeated our analysis by using the major trade route data (in Germany only) complied by Evans and Brooke^41^ and Spufford^42^ (Model 1 in Table 3) for cross-validation (Model 2 in Table 3). The same conclusion was reached. In addition, we tested for the role of navigable rivers in plague transmission in Germany (Model 3 in Table 3), and the navigable rivers were shown to be less influential than trade routes (Models 1 and 2) in plague transmission, which is consistent with our results for the whole of Europe (Table 2 and Table S8). The above findings indicated that local trade routes, which are linked with and nested within the major trade routes networks, also contributed to plague distribution significantly. Yet, the absence of fine-grained local trade route databases constrained our investigation on the topic in Germany only.

**Table 3:** OLS estimates of relationship between plague outbreak and chief/local trade route and navigable river in Germany, AD1347–1760.

Model	Log (distance)	Time fixed effect	Number of obs.	F	R^2^
Chief trade route database by Evans and Brooke^41^ and Spufford^42^					
1	−1.843461*** (−0.2278522)	Yes	728	2.19	0.4595
Local trade route dataset of Germany in Holy Roman Empire time by Davies *et al*.^47^					
2	−4.930627*** (−0.1991022)	Yes	728	6.63	0.7199
Navigable rivers					
3	−3.534741*** (0.5771989)	Yes	728	1.97	0.4326

Notes. The dependent variable of model is the total number of plague reoccurrence.

***p < 0.005; **p < 0.01; *p < 0.05.

## Discussions

Prior to the Industrial Revolution, long distance human movement was mainly confined to the trading of goods along certain overland trade routes, navigable rivers, and maritime trade routes^43^. Plague was spread primarily by its rodent host or occasionally vectorized by other animal hosts. However, these hosts did not move across Europe themselves, but were transported by humans along the major trade routes. By combing data from historical trade routes and plague records, we found that the geographic pattern of plague was determined by major trade routes in early modern Europe.

**Figure 4:**
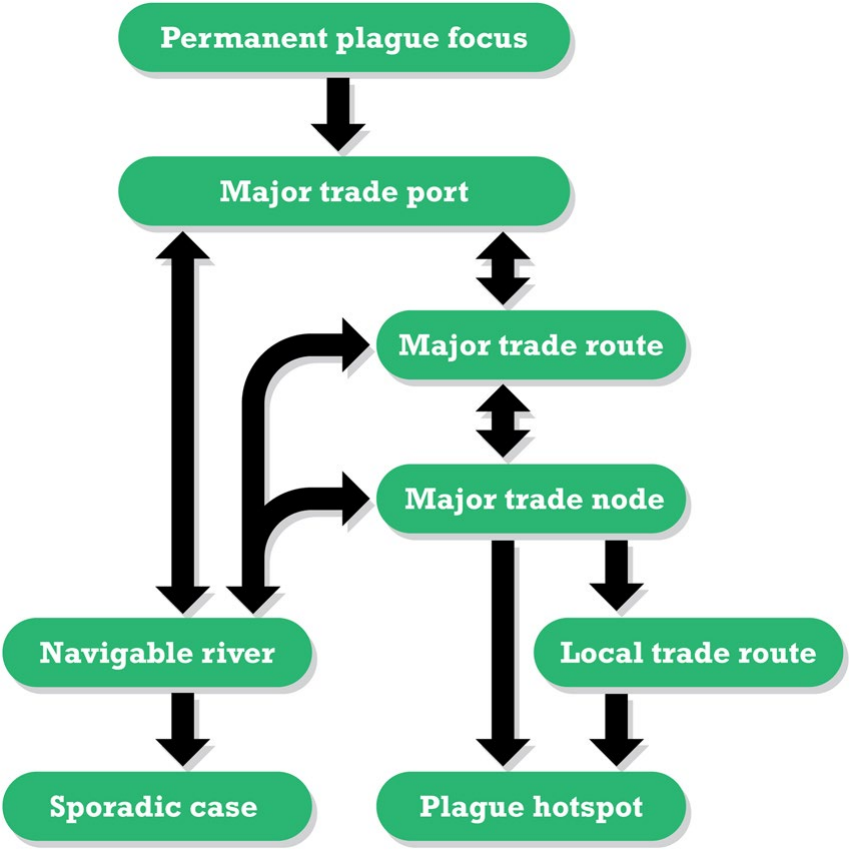
Possible plague spreading pattern from port to inland in Europe, AD1347–1760. Plagues were carried from other permanent plague focus to major trade ports in Europe. The contagion will go further to the hinterland by major trade routes or navigable river connecting the major trade ports. By transporting through the major trade route, the contagion will eventually focus at major trade node, resulting in the formation of plague hotspot in historical Europe. The pathway from major trade route to plague hotspot would also pass through local trade route. Certain amount of contagion would enter nearby navigable rivers from major trade route or major trade node. The navigable rivers would further carry the contagion inland and create sporadic cases all over the European continent.

There were five key findings in our statistical results. First, places closer to trade routes were more prone to plague outbreak and thus, plague reoccurrence. Second, plague was repeatedly introduced to several key trade ports and spread further inland in early modern Europe. Third, we found no sign of a permanent plague reservoir according to the distribution of plague outbreak. Forth, localized river navigation systems instead of trade routes accounted for the geographic distribution of sporadic plague outbreaks. Fifth, the case study of Germany suggested that local trade routes could be a significant explanatory factor to plague transmission. Based on these findings, we proposed a hypothetical corollary for plague transmission in historical Europe (Fig. 4).

Concurring with the work of Schmid *et al*.^30^, we found no evidence for a permanent plague focus in the inland of Europe. Thus, our corollary started with the assumption that plague must have been imported from a permanent plague focus to/at major trade ports. The disease probably rode on its animal hosts and approached key maritime trade ports or inland trade ports by ship. These urbanized ports and commercial hubs provided perfect conditions, such as grain warehouses, high humidity, and dense human settlements, for the establishment of epidemic epi-centers. The contagion would then pass on to major trade nodes through major trade routes. Those major trade nodes which linked up multiple trade routes would have a higher probability to become plague hotspots, as they were often connected with infected ports, or they were the infected ports themselves. Also, contagion would be likely to stop at these trade nodes, transforming them as additional epi-centers. As described by Benedictow^44^ in his work about plague transmission on the Silk Road, infected caravans would stop in caravanserai, transmitting plague to other caravans and distributing the disease across trade routes. The same phenomenon would probably apply to historical Europe. These commercial hubs would be plague hotspots and the disease was further disseminated through major trade routes such as navigable rivers, overland routes, or routes across coastal ports. A piece of supporting evidence to this plausible plague transmission mechanism was that in historical Europe, cities were small and there were no suburbs linking cities together^43^. Probably, only major trade routes would provide enough contagion density to sustain plague transmission. For other routes that were poorly built for transportation, infected people would barely survive the trip^29^. Plague would follow these major trade routes and spread to the periphery. As such, cities closer to these trade routes would have a bigger chance of infection.

It might be argued that maritime trade routes were not the sole way for plague to conquer the European continent. Plague might be repeatedly introduced to Europe through the overland trade routes such as the Silk Road. In such case, a plague pandemic would transfer from inland to port and then it was exported to other places of Europe. However, this plague transmission pathway could not be supported by our statistical results. Our robustness check showed that plague was either imported to Europe by maritime trade routes or a specific trade port was developed as a plague reservoir.

According to our results, major trade routes did not account for sporadic plague cases. In fact, the imperative role of navigable rivers in connecting cities after the medieval era has been mentioned by Edwards and Hindle^45^ and Jones^46^ and is also highlighted by our statistical analyses in spreading plague. Inland waterways provided further penetration for the contagion from various major trade routes to the hinterland. Our results proved that the further away from navigable rivers, the number of plague outbreak dropped in a statistically significant manner. Certain contagions might have left the major trade routes and spread to other settlements through inland waterways. Although these sporadic cases seemed to be randomly distributed, they were indeed anchored with trade routes.

The above explanation might not totally account for the pattern of sporadic plague outbreak in historical Europe as indicated by the low F and R^2^ values in the statistical analysis (F = 6.13, R^2^ = 0.0748, Table S7). Certain sporadic cases in our database would be attributable to other factors such as war or undocumented localized trade/communication routes. Also, sporadic events which cannot be addressed by our research methods might actually cluster in certain temporal and spatial scales. However, when we looked at long distance travel or major carriers of plague in a long-temporal and large-spatial perspective, major trade route largely accounts for the distribution of plague outbreaks.

Ideally, we would prefer to test our model with local trade route at high spatial resolution in Europe against plague distribution. Unfortunately, these data are unavailable at the moment. Given this shortcoming, we were only able to issue a case study of Germany as evidence of the role of local trade routes in plague transmission. The result further validated the possible linkage between trade route and plague recurrence, indicating that trade routes in higher resolution and local context were connected closer to plague outbreak. One possible explanation for this was that plague entered these local trade routes through the major trade route network, although it remained unclear whether this explanation could be applied in other countries.

To sum up, this study illustrated a plausible pathway for plague transmission in Europe in AD1347–1760. It had important implications in explaining how the plague outbreak pattern was shaped, and how the plague hotspots were generated, by major trade routes. The sign of permanent plague reservoirs in the inland of historical Europe could not be substantiated with strong evidence. Yet, it remains possible, according to the statistical result, that a plague reservoir once existed at the trade ports. The correlation between navigable rivers and sporadic plague outbreak cases might supplement the current explanation of plague distribution, which might provide new insight in examining the geographic patterns of plague. We did not exclude other plausible explanations for plague distribution, as there might be other factors favoring or hindering plague outbreaks in different temporal and spatial domains. Future works should focus on whether our proposed mechanism behaves differently in other spatio-temporal settings. Also, assessing whether a clustering effect in time or space exists might help explain sporadic cases, which is useful in forecasting future plague outbreaks. Most importantly, the case study result of Germany augments the high resolution trade route dataset in explaining more of the relationship between trade route and infectious disease in history. Our findings may contribute to plague prevention and mitigation, especially in the Third World where living conditions and transportation means are similar to those in pre-industrial Europe. Both major transportation routes and navigable waterways are high risk areas to be closely monitored.

## Methods

### Data

Our geo-referenced trade routes data were retrieved from the Old World Trade Routes Project built by Ciolek^24^. We combined the datasets of Evans and Brooke^41^ and Spufford^42^ to reconstruct the major trade route network from late medieval Europe to early modern time. The local trade route in Germany is originated from the study of Davies *et al*.^47^. Our geo-referenced plague outbreak data came from the database of Büntgen *et al*.^25^. Their work was originated from the literature review done by Biraben^48^. The geo-referenced database also provided coordinates for latitude and longitude of each plague outbreak case. Elevation data came from measurement through ArcGIS. Vegetation cover data of historical Europe come from the study of Kaplan *et al*.^49^. Population density was calculated from dividing the historical demographic figure from McEvedy and Jones^50^ by current regional area^51^. It was further normalized according to the data of historical urban area^49^. We retrieved the per capita Gross Domestic Product (GDP) data for our study area from the database of Bolt and Zanden^52^ and Maddison^53^. Consumer Price Index (CPI) and normal laborer’s wages were obtained from the study of Allen^54^. North Africa indicator included plague outbreaks that happened in North Africa and Turkey. Coastal indicators referred to plague outbreaks that happened at a point less than 5 km to the current coastline. Data on distance of river were acquired by measurement in ArcGIS. The definition of navigable river was based on the study of McGrail^55^ and Eckoldt^56^ and therefore we only included rivers wider than 5 m and those rivers that have a connection with other cities. We only measure rivers within a 10 km radius of plague outbreak points. For those cases where rivers were too small or too far away from plague outbreak points, 10,000 m was manually set as the distance. Please refer to SI Text for the details of our datasets.

### Ordinary Least Square (OLS) estimation

We hypothesize the pattern of plague outbreak to be determined by trade routes. To validate the robustness of the above relationship, we include various control variables in our regression models. Our base regression model is:

1 $$\,{P}_{c}=\propto +\beta (log{D}_{i})+{{\mathscr{X}}}_{i}^{\text{'}}\delta +{C}_{i}^{\text{'}}\varphi +{\varepsilon }_{i},\,$$

where *P*_*c*_ is the total number of plague count at an individual point, $\propto$ is the intercept of the equation, $\beta$ is the coefficient of the association, $log{D}_{i}$ stands for the logged distance to the closest major trade route from each individual plague outbreak point, ${{\mathscr{X}}}_{i}^{\text{'}}$ is the vector of controlled variables in the model, ${C}_{i}^{\text{'}}$ is the country fixed-effects estimator that controls the differences among regions, ${\varepsilon }_{i}$ is the error term. Time fixed-effects estimator is only added to those models with time variants. The relationship between trade route and plague outbreak remains significant even if the time-fixed effects estimator is excluded. The above method is also applied in the remaining parts of our statistical analyses.

### Robustness checks

We perform various robustness checks to validate the significance of our results in different temporal and spatial domains. We built different regression models to explore the impact of trade routes on plague outbreak patterns in different regional settings. This helps to avoid the potential regional discrepancies that are not controlled in our models. Furthermore, factors such as technological improvement are hard to quantify, but they are related to the performance of trade routes over time. Therefore, we slice our data into different time sections to see whether trade routes were still significant in shaping plague outbreak pattern over time. The results of these robustness checks are presented in the Appendix.

## Electronic supplementary material

Supplementary information
