# Supplementary material for: Trade routes and plague transmission in pre-industrial Europe
Source: Sci Rep. 2017 Oct 11;7:12973. doi: 10.1038/s41598-017-13481-2 (PMC5636801; doi:10.1038/s41598-017-13481-2)
Supplement: Supplementary file 1 — Supplementary information [file 41598_2017_13481_MOESM1_ESM.doc]

**Trade routes and plague transmission in pre-industrial Europe**

Ricci P.H. Yue ^a*^, Harry F. Lee ^a, b*^, Connor Y.H. Wu ^c^

^a^ Department of Geography, The University of Hong Kong

^b^ International Center for China Development Studies, The University of Hong Kong

^c^ Department of Population Health Sciences, Virginia-Maryland College of Veterinary Medicine, Virginia Tech

^*^ Corresponding author. Tel.: +852 6504 0880; Fax: +852 2559 8994.

E-mail address: [ricciyue@connect.hku.hk](mailto:ricciyue@connect.hku.hk) (R.P.H. Yue), [harrylee@hku.hk](mailto:harrylee@hku.hk) (H.F. Lee)

**Table S1.** Summary statistics of the variables used in our OLS estimates.

**Table S2.** Linear correlation between frequent plague outbreak point and trade node/port

**Table S3.** Robustness check for the plague/trade route relationship in different temporal and spatial domains

**Table S4**. OLS estimates of relationship between plague outbreak and port location in Europe, AD1347 - 1760

**Table S5.** Robustness check for the plague/port relationship in different temporal and spatial domains

**Table S6.**  Evaluation of trade route performance on sporadic plague outbreak

**Table S7.** Comparison of performance between trade route and navigable river in explaining sporadic outbreak

**Table S8.** OLS estimates of relationship between plague outbreak and trade route in Europe, AD1347 - 1760

**Figure S1.** Spatial distribution of plague outbreak in Europe in different periods. The frequency of plague reoccurrence is corresponding to the size of dots. (A). AD1347 – 1760. (B). AD1347 – 1449. (C). AD1450 – 1549. (D). AD1550 – 1649. (E). AD1650 – 1760. The map is generated in ArcGIS version 10.1 (www.esri.com/software/arcgis).

**Figure S2.** Distribution of trade route (blue line), major trade ports with plague outbreak record (black dot) and major trade ports without plague outbreak (grey dot). The size of dot refers to the frequency of plague at the port. Only trade route and trade ports at countries with plague record were shown. The map is generated in ArcGIS version 10.1 (www.esri.com/software/arcgis).

**Figure S3.** Distribution of sporadic plague cases (frequency <5) in historical Europe, AD1347-1760. The map is generated in ArcGIS version 10.1 (www.esri.com/software/arcgis).

**Supplementary text**

**Detailed material and methodology**

Our study focused on a period from AD1347 to AD1760. The starting year referred to the earliest available record in the plague dataset we applied in this study. It is also the year that plague was introduced to Italy which turned out killing almost half of the population in Europe in the next decade^1^. AD1760 was considered as the watershed between the pre-industrial era and industrial revolution^2^. Probably due to the advance in sanitation and technology, the number of plague case declined quickly in Europe after 1760^3^. Yet, there was no unanimous conclusion from academia to explain such pattern. Therefore, our model mainly focused on the pre-industrial era. The majority of the plague outbreak was recorded in European continents. Certain locations in North Africa, Russian region and European Turkey were able to provide records to the plague database. Our study area, therefore, focused on Europe and North Africa.

**Plague data**

In the previous study of Ricci Yue, et al^. 4^, they excluded all plague incidences at the 5km proximity of the coast since they only focused on inland plague outbreaks. However, we included both coastal outbreak and inland outbreak in this study so that we could cover every plague outbreak within our study period based on the database constructed by Büntgen, et al^. 3^. The database incorporated a total of 6656 plague outbreaks from AD1347 to AD1760 over the European continents and Northern Africa. We combined multiple outbreaks in the same year as one outbreak since there was no indication of the starting time and ending time of outbreak. Yet, it is reminded to the readers one shortcoming of the dataset is that the count of plague outbreak does not necessarily represent the scale of outbreaks. Also, outbreaks that occurred during the end of the year/beginning of the new year were listed twice in the original database. For example, in London, literature suggested that the city has experienced 5–6 major plague strikes within the time of AD 1500–1665 ^5^. The magnitude of these strikes was hard to quantify and the continued plague the year following the major epidemic was separated in the dataset. Therefore, the count of plague outbreak synthesizes only the number of year of plague outbreak.

**Trade route**

In our study we had taken account the impact of both maritime trade route and overland trade route (Figure S2). The major port locations and major maritime trade routes were retrieved from the studies by Spuffor^d 6^ and Evans and Brook^e 7^that indicated the pattern of maritime trading at early modern period. The overland trade route was retrieved from the work of Evans and Brook^e 7^ that summarized the chief trade routes of early modern period. The trade route database was derived from data provided on the website of Old World Trade Routes Project (http://www.ciolek.com/owtrad.html) by Ciole^k 8^. The dataset covered major European countries and also their linkage to Northern Africa. In addition to the port cities indicated from the study by Spuffor^d 6^. Trade nodes at the 5km periphery of the coastline were also regarded as major port cities. These dataset covered a vast area but we only included ports and routes that have connections to countries with plague record. One important step in revealing the extent of impact of trade route is to include the localized trade route in the estimation. We achieve this by using the dataset provided by Davies, et al^. 9^ that mapped out the localized trade route of Germany in Holy Roman Empire period.

**Coordinates**

The spatial information of plague outbreak points could also serve as rough estimation for climate zone and tendency of distribution. For latitude, basically it is hotter towards the equator, and vice versa. For longitude, it contains information of whether plague outbreak inclined to the side of Western Europe or Eastern Europe. The coordinates were retrieved from the geo-reference as built in in the plague database constructed by Büntgen, et al^. 3^

**Elevation**

As concluded by the study previously from Ricci Yue, et al^. 4^, elevation is one of the determining factors for plague outbreak. According to their theory, inland spreading of plague significantly relied on good transport and human movement on navigable rivers. Thus, plague broke out mainly at navigable-river-densed regions. As such, we included this variable as a control variable to test for the robustness of river/trade route relationship. The elevation of plague outbreak point was measured by the built–in function of ArcGIS v10.1.

**Coastal indicator**

To control for the potential difference between coastal cities and inland cities, we introduced a coast indicator as a control variable. In the binary system, one stood for plague outbreak points in the 5km proximity of the coastline. Zero was donated to plague outbreak points outside the set distance. In summary, 1042 out of 6656 times of plague outbreak were regarded as coastal outbreak.

**North Africa indicator**

North Africa holds a significantly different culture than major Europe countries. Therefore, we also introduced a North Africa indicator to control for the disparity. In the system, one represented plague outbreak from North Africa and zero referred to outbreak in European continent. A total of 292 outbreaks were recorded as North Africa outbreak.

**Vegetation cover**

Vegetation cover pertained the degree of vegetation coverage on land available for agriculture^10^. As time processes, vegetation cover was removed due to urbanization and development. Therefore, degree of vegetation cover could indicate the conditions of urban environment across spatial domain and temporal domain. Our vegetation cover index was derived from the database built from Kaplan, et al^. 10^. Missing data was inserted with linear interpolation.

**Normalized population density**

The fundamental element for human plague is the presence of human. Therefore, instead of population density over the entire country, we were more interested in the ‘real’ population density over populated area. In order to normalize the historical population data constructed by McEvedy and Jone^s 11^, we borrowed the usable land data provided by Kaplan, et al^. 10^ for normalization. The dataset could better reflect the population density and also the degree of urbanization over space and time. Missing data in the historical population database was created by exponentially interpolation.

**Per capita GDP**

Per capita GDP measures the averaged economic performance of the people in a country. Therefore we introduced this control variable to assess whether economic attribute impacts on the distribution of plague outbreak over time and space. Here we adopted the historical Per capita GDP database constructed by Maddiso^n 12^ and Bolt and Zande^n 13^. Linear Interpolation was inserted in missing data. We borrowed data from the nearest country if the first data of a country appeared after 1347.

**Real wage**

Real wage refers to the adjusted income level after taking into account the effect of inflation or depletion in purchasing power over time. As described by Alle^n 14^, the real wage reflected the minimum purchasing power for the least amount of diets to sustain lives in history. Therefore the real wage also indicated the situation of the most vulnerable people at a time and also the welfare condition at the previous time^15^. The database here came from the historical real wage database for normal labourers in 19 different European countries developed by Alle^n 16^. Linear interpolation was applied for missing data. For region with their first datapoint found after 1347, we assigned data from the closest region.

**Consumer price index**

Consumer price index (CPI) here compiled information of the price of daily necessity such as wheat, barley, oat, rye, beef, peas, cheese, eggs, wax, charcoal, firewood, oil, honey, herring, sugar, beer, etc. These information were standardized and converted into a historical CPI database by Alle^n 16^. The database covered 19 cities around different parts of Europe. Contrary to per capita GDP, which represented the averaged economic output per person in a country, CPI surrogated the cost of living of the time and the averaged cost to sustain the basic living standard in different times and spaces. Missing data was inserted by linear interpolation. Country with their first data point after 1347 borrowed figures from the closest region.

**Distance to river**

Distance to river was measured from the centre of the plague outbreak point provided by Büntgen, et al^. 3^ to the closest possible navigable river. In this study, the definition of navigable river was made according to the previous study by Ricci Yue, et al^. 4^. In short, the size of fleet in history was taken into account for deciding ‘navigable’. Also, the water body needed to have connection with other city. It had to be noted some cities were surrounded by multiple water systems and we only took the one closest distance. If no navigable river was detected within the 10km periphery of the plague outbreak point, 10km was automatically donated as dummy value.

**Sensitivity check for frequent plague outbreak points-trade nodes/trade ports relationship**

Table S2 showed the result of sensitivity check to indicate that frequent plague outbreak points are likely to be key trade nodes, but not likely to be major trade ports. Linear regression was applied. Sporadic plague outbreaks (frequency <5) were excluded in this analysis. From the reported results, the null hypothesis that plague outbreak point with more outbreaks was not key trade nodes was defeated. On the other hand, we had no proof to say that key trade ports were plague hotspots.

**Robustness check for plague/trade route relationship**

Table S3 showed the result of sensitivity check of plague/trade route relationship in different spatial or temporal specification. We applied OLS estimates for analyzing the data. Only regional fixed effect was inserted in the models for there is no time variant in any of the specifications. In Check A to D, we dissected our study period into four roughly 100-year intervals (AD1347-1449; AD1450-1549; AD1550-1649; AD1650-1760). We recalculated the total number of plague outbreak at a given point according to the duration of the check. The results were all highly significant and negative with a range of R^2^ of from 0.2736 to 0.8170. The F-value ranged from 39.35 to 179.87. Based on the result, no linear pattern across time could be found. In Check E to H, we checked the robustness of plague/trade route relationship in different geographical setting. Check E dropped all plague outbreaks from North Africa, for the potential disparity of plague source from African continent. In Check F, we further dropped plague outbreak in Russian area too. The model in Check F avoided data from Far East and African region, therefore it could be deemed as a focused study on Europe. In Check G the plague outbreak in UK was further excluded from the specification since it was out of major continent of Europe. In Check H we selected plague outbreak of the top six plague outbreak countries (UK, France, Italy, Germany, Spain and Portugal). They combined for 86.67% of the total plague outbreak recorded in the database. In the four specifications that displayed at Check E to Check G, the relationship of plague/trade route remained highly significant and negative with a range of F-value from 219.39 to 446.48. The R^2^ ranged from 0.3178 to 0.3327. The results from Check A to Check H showed that the relationship between plague outbreak and trade route was robust and negative and highly significant in every specification.

**Robustness check for plague/port relationship**

The sensitivity check for plague/port relationship was shown in Table S4. The aim of the check was to test for the robustness of the relationship in different spatial and temporal specifications. OLS estimate was applied for the robustness with regional fixed effect included in every specification. The dependent variable was the total number of plague outbreak at a given point during the corresponding study period of each check. For Check A to D, we separated our study period into a roughly 100-year interval, e.g. 1347-1449; 1450-1549; 1550-1649; 1650-1760). The result showed that plague/port relationship was highly significant and negative in all specifications. The F-value ranged from 11.99 to 197.58. The R^2^ ranged from 0.1902 to 0.8306. For Check E to J, different spatial specifications were introduced to the OLS estimates. In Check E, the plague incidences in African continents were excluded. In Check F, plague outbreaks in Russian region were also dropped from the model. The African region and Russian region were dropped because they are far from the major European outbreak during our study period. In Check G, we further trimmed off UK from the model so that only continental outbreak was included. In Check H, we selected outbreaks from the top six plague outbreak countries (France, Germany, Italy, Portugal, Spain and UK) only. In Check I and J, we divided outbreak into Mediterranean Europe and Central/Northern Europe ^17^ to isolate the regional difference between the N/S Europe. For Check E to J, which specified different spatial settings, the relationship between port and river remained statistically significant and negative. The F-value ranged from 87.5 to 283.68. The R^2^ ranged from 0.1576 to 0.3896. In short, the association under investigation was robust and highly significant in different temporal and spatial settings. The result approved our hypothesis that more plague outbreak happened when distance to major trade port reduced in pre-industrial Europe.

**Evaluation of trade route performance on sporadic plague outbreak**

We evaluated the performance of trade route in explaining the extent of sporadic plague outbreak cases as shown in Figure S7. In the sensitivity check we gradually decreased the accumulated number of recurrence to cross check the efficiency of the explanatory power of trade route as the independent variable to plague recurrence. Judging from the F-value and R^2^ value, the explanatory power of trade route decreased with plague recurrence time. However, distance from trade route remained as a significant determining factor with plague recurrence time >5 within our study period. However, based on the statistical result, distance from trade route was not able to correlate significantly with plague recurrence when the recurrence frequency dropped to 4 times or less than 4 times. It indicated that distance of trade route could not explain the distribution of these sporadic plague outbreak cases.

| Table S1 | | | | | |
| --- | --- | --- | --- | --- | --- |
| Summary statistics of the variables used in our OLS estimates | | | | | |
| Variable | Mean | Std. dev. | Min | Max | N |
| Count of plague outbreak | 27.39 | 27.73 | 1 | 132 | 6656 |
| Log(distance from trade route) | 3.17 | 2.13 | 0 | 5.67 | 6656 |
| Log(distance from port) | 4.51 | 1.77 | 0 | 5.94 | 6656 |
| Distance from equator (degree) | 47.21 | 4.89 | 30.08 | 60.37 | 6656 |
| Longitude(degree) | 4.57 | 6.90 | -9.26 | 37.55 | 6656 |
| Elevation (m) | 134.30 | 166.27 | 0 | 1149 | 6656 |
| Coast indicator | 0.16 | 0.36 | 0 | 1 | 6656 |
| North Africa indicator | 0.044 | 0.20 | 0 | 1 | 6656 |
| Vegetation cover (%) | 18.62 | 10.32 | 0.64 | 65 | 6656 |
| Normalized population density (no. of person/km^2^) | 455.08 | 835.59 | 6.82 | 10481.17 | 6656 |
| Per capita GDP (1990 Int. GK$) | 1038.28 | 234.90 | 365.54 | 1753.18 | 6656 |
| Real wage (grams Ag/day) | 4.77 | 1.34 | 0.57 | 9.11 | 6656 |
| Consumer price index (Ag price) | 0.98 | 0.46 | 0.13 | 2.67 | 6656 |
| Log(Distance to river) (km) | 2.72 | 0.75 | 0 | 4 | 6656 |

| Table S2 | | | |
| --- | --- | --- | --- |
| Linear correlation between frequent plague outbreak point and trade node/port | | | |
|  | Coef | F | R^2^ |
| Key trade node | 14.45512 | 44.95*** | 0.13 |
| Major trade port | 3.943288 | 2.37 | 0.01 |

Note: n = 307. *** p<0.005

| Table S3 | | | | | |
| --- | --- | --- | --- | --- | --- |
| Robustness check for the relationship between trade route and plague outbreak | | | | | |
| Check | Width of River | Regional fixed effect | Number of obs. | F | R^2^ |
| First part: Sensitivity check across temporal domain | | | | | |
| A. Using only plague outbreak during AD1347-1449 | | | | | |
| Period count | -2.160663***  (-0.6064948) | Yes | 938 | 39.35 | 0.4352 |
| B. Using only plague outbreak during AD1450-1549 | | | | | |
| Period count | -2.735334***  (-0.432121) | Yes | 1871 | 62.00 | 0.3486 |
| C. Using only plague outbreak during AD1550-1649 | | | | | |
| Period count | -1.956432***  (-0.3459891) | Yes | 3119 | 68.71 | 0.2736 |
| D. Using only plague outbreak during AD1650-1760 | | | | | |
| Period count | -0.3405505**  (-0.0525955) | Yes | 703 | 179.87 | 0.8170 |
| Second part: Sensitivity check across spatial domain | | | | | |
| E. Dropping Africa | | | | | |
|  | -6.671448***  (-0.5113197) | Yes | 6364 | 219.39 | 0.3260 |
| F. Dropping Russia and Africa | | | | | |
|  | -6. 910207***  (-0.5209806) | Yes | 6063 | 251.31 | 0.3327 |
| G. Only Continental Western Europe (without UK) | | | | | |
|  | -5.240929***  (-0.4653725) | Yes | 4879 | 266.24 | 0.3298 |
| H. Major 6 countries (France, Germany, Italy, Spain, Portugal and UK) | | | | | |
|  | -6.880653***  (-0.5066506) | Yes | 5759 | 446.49 | 0.3178 |

Notes. The dependent variable of checks is the total number of plague reoccurrence within each corresponding period.

*** p <0.005； ** p <0.01； * p <0.05

| Table S4 | | | | |
| --- | --- | --- | --- | --- |
| OLS estimates of relationship between plague outbreak and port location in Europe, AD1347-1760 | | | | |
| Log (portD) | Regional fixed effect | Number of obs. | F | R^2^ |
| Log(Distance to port) | | | | |
| -6.740251*** (-0.4312615) | yes | 6656 | 114.38 | 0.2658 |

*** p<0.005

| Table S5 | | | | | |
| --- | --- | --- | --- | --- | --- |
| First part: Sensitivity check across temporal domain | | | | | |
| Check | Log (portD) | Time/Regional fixed | Number of obs. | F | R^2^ |
| A. Using only plague outbreak during AD1347-1449 | | | | | |
| Period count | -1.427044***  (-0.3543612) | N/Yes | 938 | 11.99 | 0.1902 |
| B. Using only plague outbreak during AD1450-1549 | | | | | |
| Period count | -2.595951***  (-0.3293006) | N/yes | 1871 | 40.98 | 0.2612 |
| C. Using only plague outbreak during AD1550-1649 | | | | | |
| Period count | -2.363692***  (3559-0.34) | N/yes | 3119 | 63.73 | 0.2589 |
| D. Using only plague outbreak during AD1650-1760 | | | | | |
| Period count | -1.050608***  (-0.1601872) | N/yes | 703 | 197.58 | 0.8306 |
| Second part: Sensitivity check across spatial domain | | | | | |
| E. Dropping Africa | | | | | |
|  | -6.883567***  (-0.4165264) | N/yes | 6364 | 138.40 | 0.2338 |
| F. Dropping Russia and Africa | | | | | |
|  | -6.977304***  (-0.4228252) | N/yes | 6063 | 154.97 | 0.2351 |
| G. Only Continental Western Europe (without UK) | | | | | |
|  | --3.096006***  (-0.2063985) | N/yes | 4879 | 102.86 | 0.1576 |
| H. Major 6 countries (France, Germany, Italy, Spain, Portugal and UK) | | | | | |
|  | -7.105249 ***  (-0.4221001) | N/yes | 5759 | 283.68 | 0.2283 |
| I. Mediterranean Europe | | | | | |
|  | -1.87289***  (-0.1613317) | N/yes | 1520 | 87.50 | 0.3896 |
| J. Central/Northern Europe | | | | | |
|  | -10.37481***  (-0.5341659) | Yes | 5136 | 188.20 | 0.3060 |

Notes. The dependent variable of checks is the total number of plague reoccurrence within each corresponding period.

*** p <0.005； ** p <0.01； * p <0.05

| Table S6 | | | | | | | |
| --- | --- | --- | --- | --- | --- | --- | --- |
| Evaluation of trade route performance on sporadic plague outbreak | | | | | | | |
| Sporadic Case (count <) | 10 | 8 | 6 | 5 | 4 | 3 | 2 |
| N | 2404 | 2113 | 1743 | 1563 | 1383 | 1119 | 891 |
| F | 28.15 | 19.91 | 10.60 | 10.47 | 5.54 | 2.49 | 3.89 |
| Coef. (beta) | -0.5978516***  (-0.2542766) | -0.5819441***  (-0.2867905) | -0.2647711***  (-0.1458994) | -0.1251746**  (-0.080018) | -0.284492  (-0.0215463) | 0.0254784  (0.0269838) | -0.0141177  (-0.024579) |
| R^2^ | 0.183 | 0.146 | 0.100 | 0.109 | 0.068 | 0.039 | 0.074 |

Notes. The dependent variable of evaluation is the defined number of plague reoccurrence for sporadic outbreak. The predictor is the log(distance to major trade route)

*** p <0.005； ** p <0.01； * p <0.05

| Table S7 | | | | | |  | | | | | | | |
| --- | --- | --- | --- | --- | --- | --- | --- | --- | --- | --- | --- | --- | --- |
| Comparison of performance between trade route and navigable river in explaining sporadic outbreak | | | | | |  | | | | | | | |
|  | Log(Distance to trade route) | Log(Distance to river) | Without Africa | Without Africa, Russia | Major countries |  | | | | | | | |
| Sporadic case (count <) | 4 | 4 | 4 | 4 | 4 |  | | | | | | | |
| N | 1383 | 1383 | 1370 | 1357 | 1197 |  | | | | | | | |
| F | 5.54 | 6.13 | 8.08 | 8.43 | 12.96 |  | | | | | | | |
| Coef. (beta) | -0.284492  (-0.0215463) | -0.027214***  (-0.090248) | -0.0267***  (-0.0876226) | -0.0264***  (-0.0861262) | -0.0251**  (-0.0816937) |  | | | | | | | |
| R^2^ | 0.068 | 0.075 | 0.072 | 0.070 | 0.061 |  | | | | | | | |
|  |  |  |  |  |  |  |  |  |  |  |  |  |  |

Notes. The dependent variable of model is the total number of plague reoccurrence within the sporadic plague outbreak model.

*** p <0.005； ** p <0.01； * p <0.05

| Table S8 | | | | |
| --- | --- | --- | --- | --- |
| OLS estimates of relationship between plague outbreak and trade route in Europe, AD1347 - 1760 | | | | |
| Log (distance to navigable river) | Time/Regional fixed | Number of obs. | F | R^2^ |
| -5.860268***  (-0.1595181) | Yes/Yes | 6656 | 5.18 | 0.2586 |

Notes. The dependent variable of model is the total number of plague reoccurrence.

*** p <0.005； ** p <0.01； * p <0.05

**
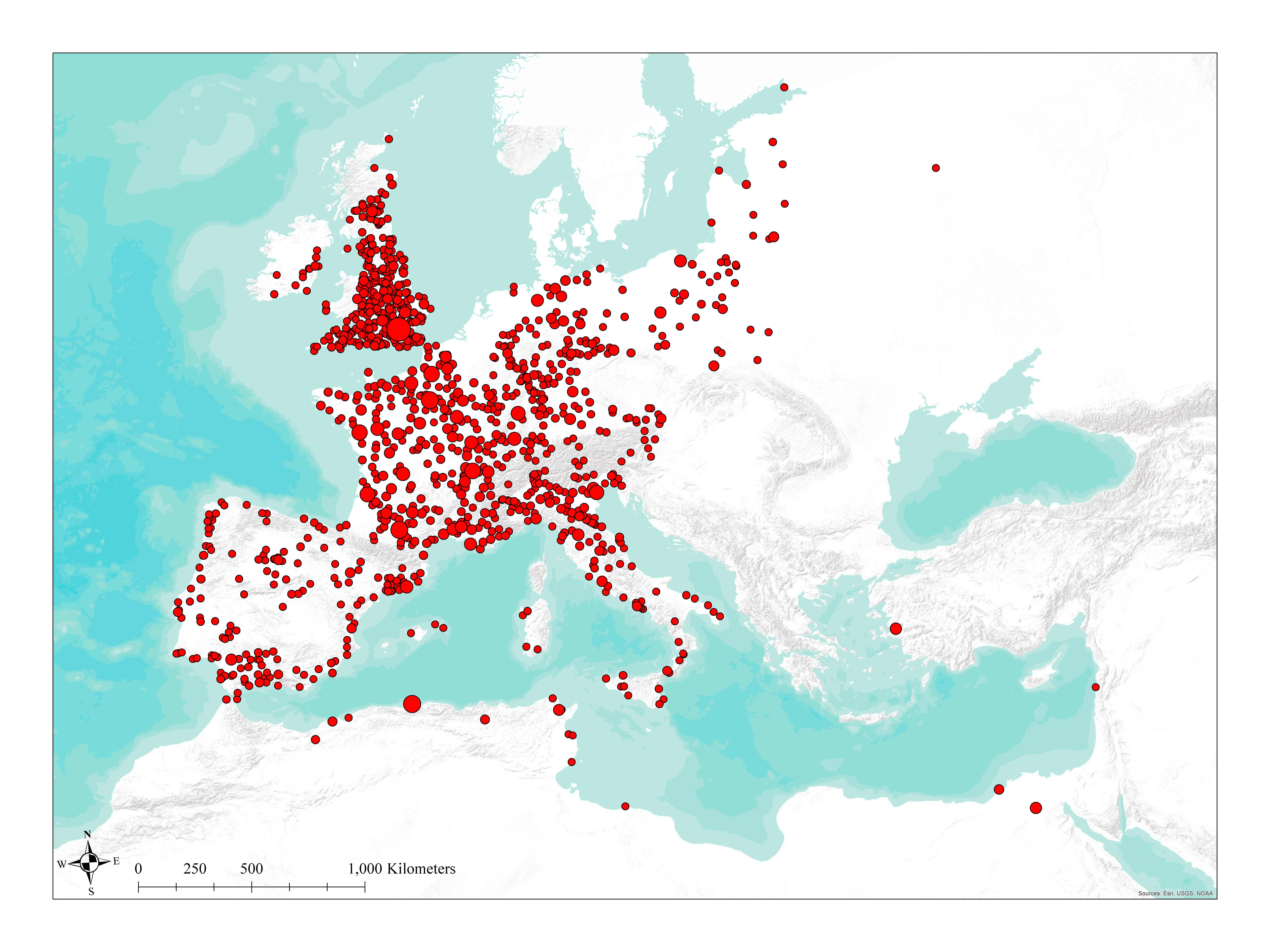
**

**(A)**

**
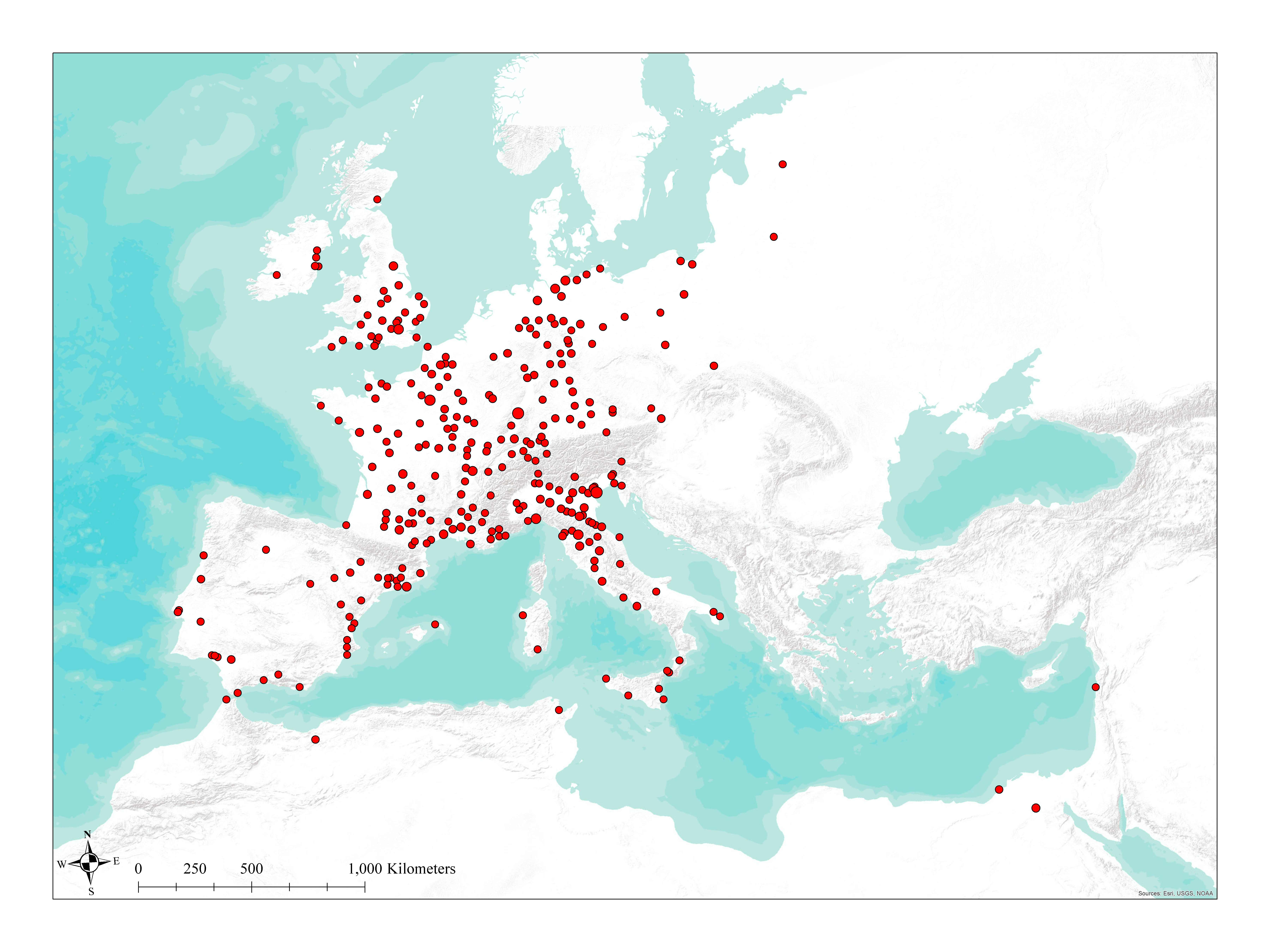
**

**(B)**

**
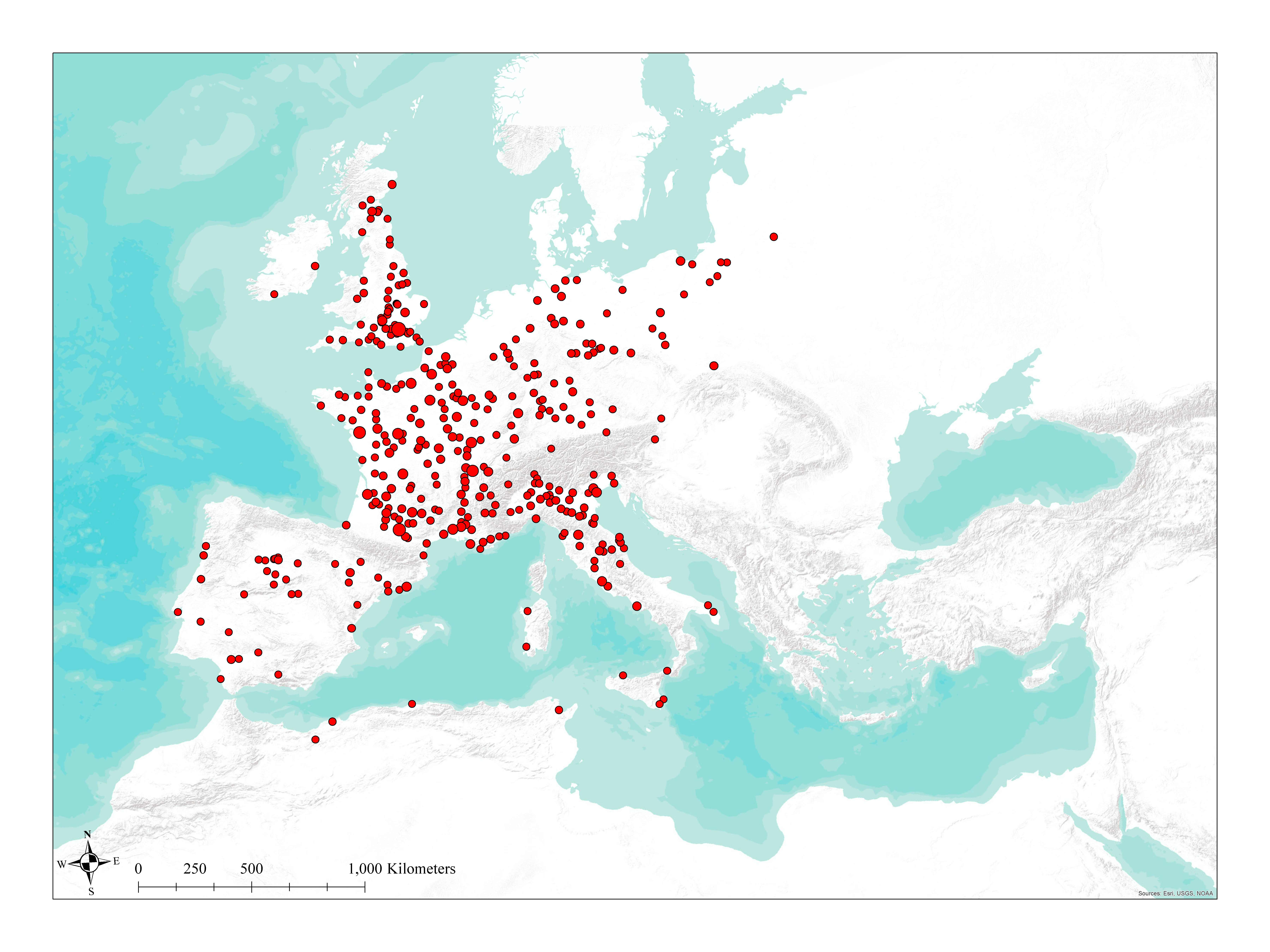
**

**(C)**

**
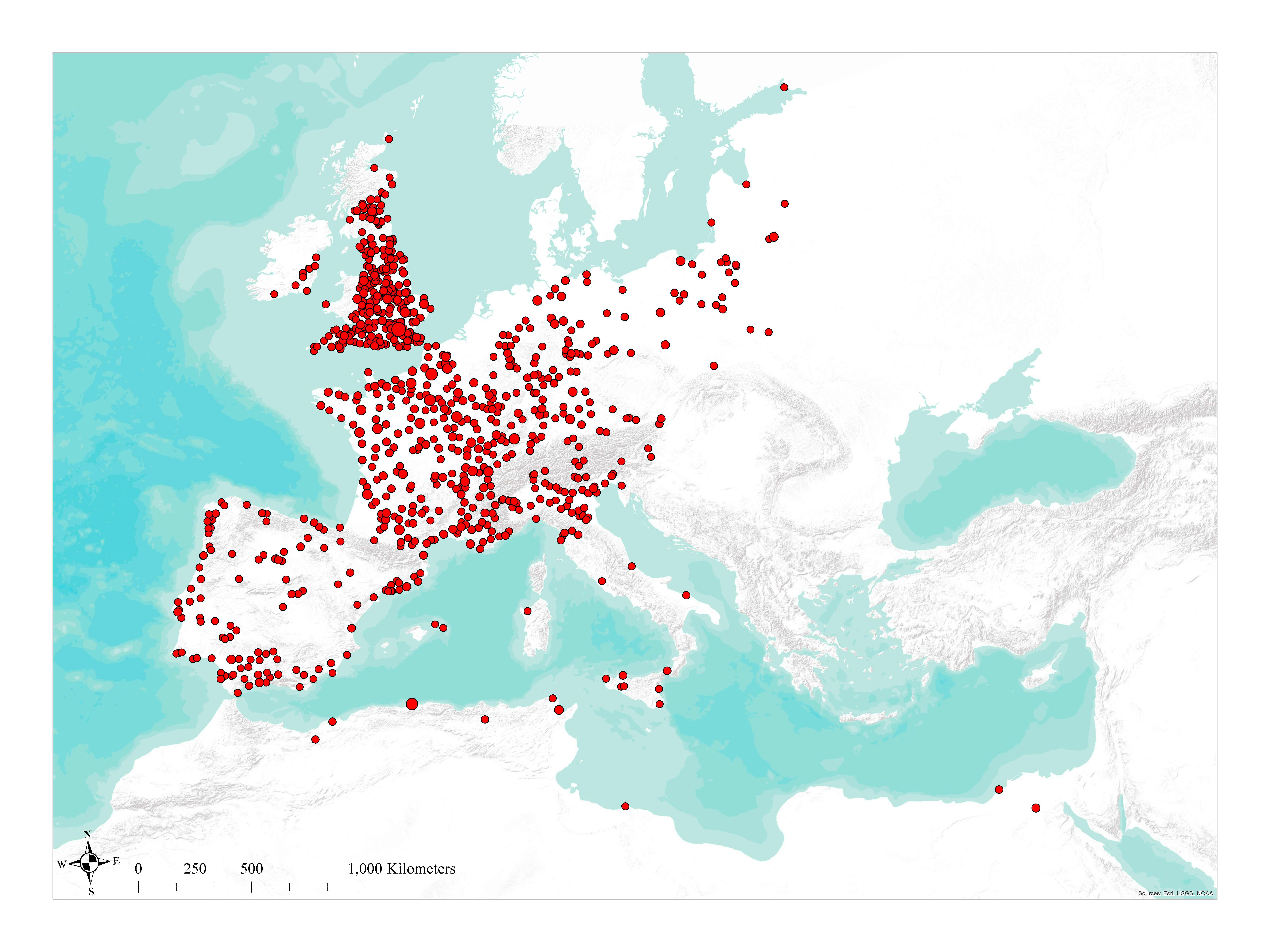
**

**(D)**

**
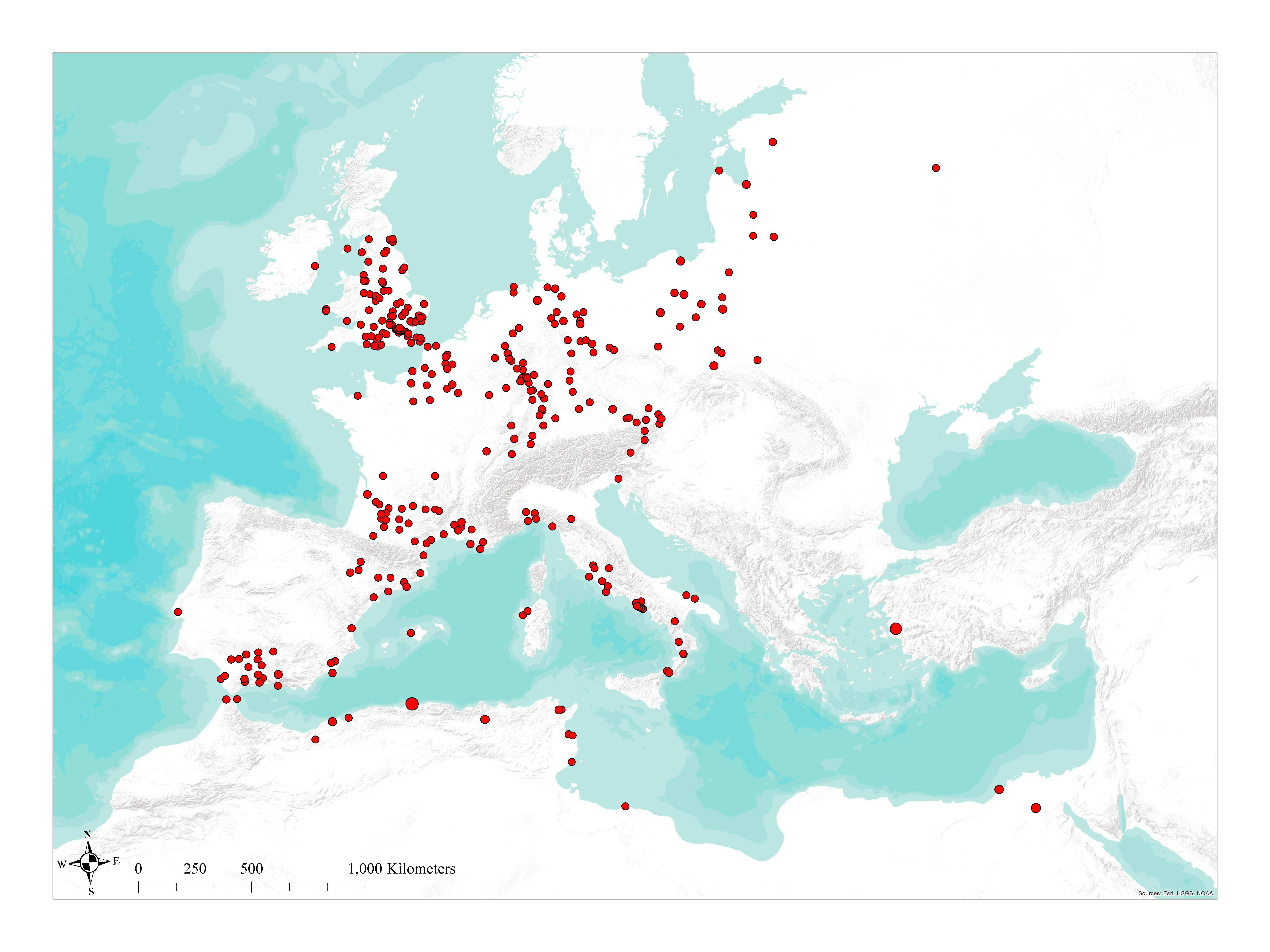
**

**(E)**

**Figure S1.** Spatial distribution of plague outbreak in Europe in different periods. The frequency of plague reoccurrence is corresponding to the size of dots. (A). AD1347 – 1760. (B). AD1347 – 1449. (C). AD1450 – 1549. (D). AD1550 – 1649. (E). AD1650 – 1760. The map is generated in ArcGIS version 10.1 (www.esri.com/software/arcgis).


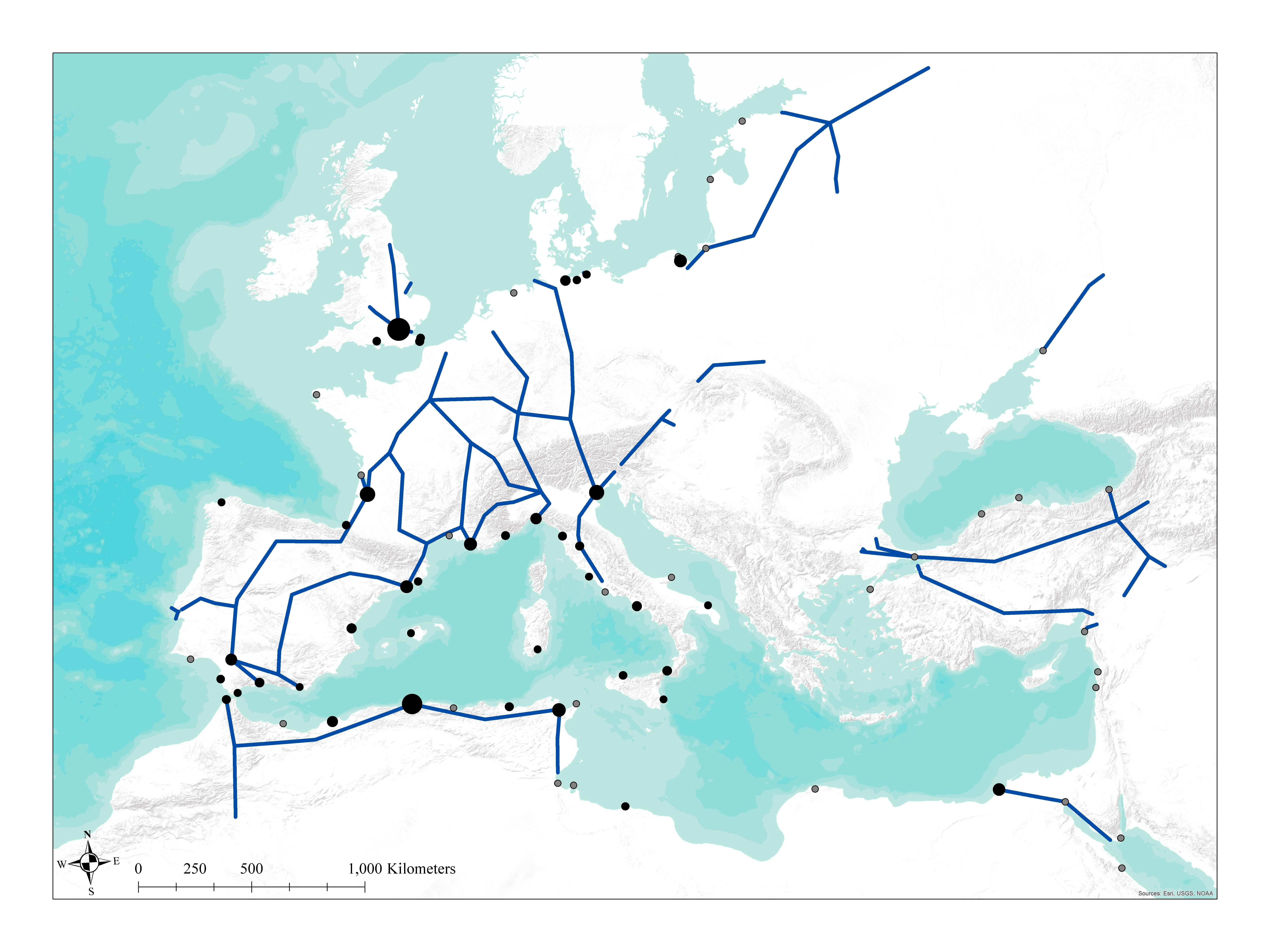


**Figure S2.** Distribution of trade route (blue line), major trade ports with plague outbreak record (black dot) and major trade ports without plague outbreak (grey dot). The size of dot refers to the frequency of plague at the port. Only trade route and trade ports at countries with plague record were shown. The map is generated in ArcGIS version 10.1 (www.esri.com/software/arcgis).

**
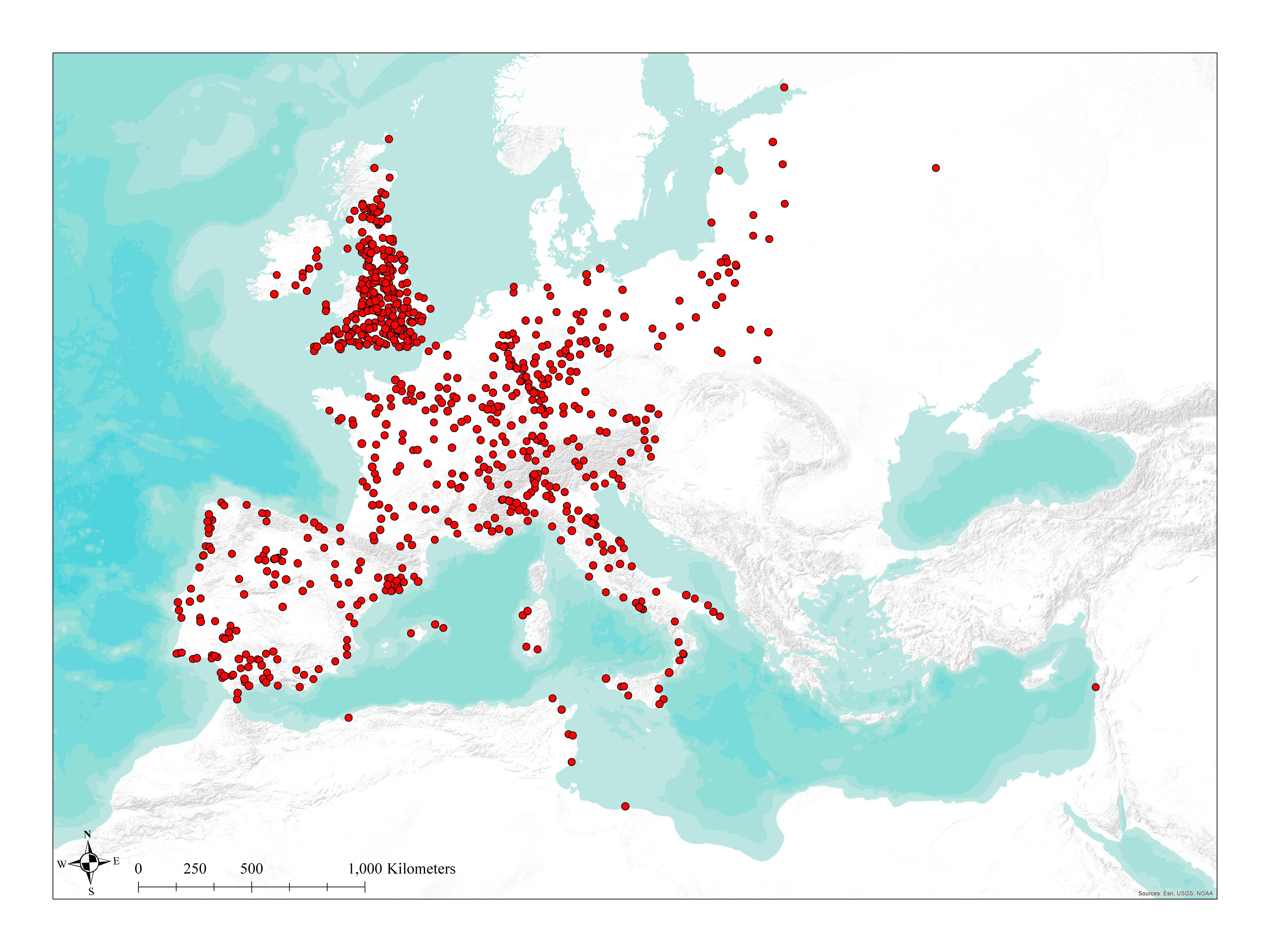
Figure S3.** Distribution of sporadic plague cases (frequency <5) in historical Europe, AD1347-1760. The map is generated in ArcGIS version 10.1 (www.esri.com/software/arcgis).

References:
